# Influence of TiO_2_ and ZnO Nanoparticles on α-Synuclein and β-Amyloid Aggregation and Formation of Protein Fibrils

**DOI:** 10.3390/ma15217664

**Published:** 2022-10-31

**Authors:** Nora Slekiene, Valentinas Snitka, Ingrida Bruzaite, Arunas Ramanavicius

**Affiliations:** 1 Pharmacy Center, Institute of Biomedical Sciences, Faculty of Medicine, University of Vilnius, M.K. Čiurlionio g. 21/27, LT-03101 Vilnius, Lithuania; 2 Research Center for Microsystems and Nanotechnology, Kaunas University of Technology, 65 Studentu Str., LT-51369 Kaunas, Lithuania; 3 Department of Chemistry and Bioengineering, Faculty of Fundamental Sciences, Vilnius Gediminas Technical University, Sauletekio Av. 11, LT-10223 Vilnius, Lithuania; 4 Laboratory of Electrochemical Energy Conversion, State Research Institute Centre for Physical Sciences and Technology, Sauletekio Av. 3, LT-10257 Vilnius, Lithuania; 5 Department of Physical Chemistry, Faculty of Chemistry and Geosciences, Vilnius University, 24 Naugarduko Str., LT-03225 Vilnius, Lithuania; 6 Laboratory of Nanotechnology, State Research Institute Centre for Physical Sciences and Technology, Sauletekio Av. 3, LT-10257 Vilnius, Lithuania

**Keywords:** α-synuclein aggregation, β-amyloid fragment 1-40, TiO_2_ nanoparticles, ZnO nanoparticles, protein fibrils, surface-enhanced Raman spectroscopy (SERS), tip-enhanced Raman spectroscopy (TERS), principal component analysis (PCA), atomic force microscopy (AFM), UV-Vis spectroscopy

## Abstract

The most common neurological disorders, i.e., Parkinson’s disease (PD) and Alzheimer’s disease (AD), are characterized by degeneration of cognitive functions due to the loss of neurons in the central nervous system. The aggregation of amyloid proteins is an important pathological feature of neurological disorders.The aggregation process involves a series of complex structural transitions from monomeric to the formation of fibrils. Despite its potential importance in understanding the pathobiology of PD and AD diseases, the details of the aggregation process are still unclear. Nanoparticles (NPs) absorbed by the human circulatory system can interact with amyloid proteins in the human brain and cause PD. In this work, we report the study of the interaction between TiO_2_ nanoparticles (TiO_2_-NPs) and ZnO nanoparticles (ZnO-NPs) on the aggregation kinetics of β-amyloid fragment 1-40 (βA) and α-synuclein protein using surface-enhanced Raman spectroscopy (SERS) and tip-enhanced Raman spectroscopy (TERS). The characterizations of ZnO-NPs and TiO_2_-NPs were evaluated by X-ray diffraction (XRD) spectrum, atomic force microscopy (AFM), and UV-Vis spectroscopy. The interaction of nanoparticles with amyloid proteins was investigated by SERS. Our study showed that exposure of amyloid protein molecules to TiO_2_-NPs and ZnO-NPs after incubation at 37 °C caused morphological changes and stimulated aggregation and fibrillation. In addition, significant differences in the intensity and location of active Raman frequencies in the amide I domain were found. The principal component analysis (PCA) results show that the effect of NPs after incubation at 4 °C does not cause changes in βA structure.

## 1. Introduction

Future breakthroughs in the understanding of fundamental biological processes and molecular events causing major disease need to visualize, manipulate, modify, and characterize nanoobjects. However, the detection of protein deposits and the evaluation of their aggregation kinetics are generally based on fluorescent probes [1,2,3]. The application of label-free methods is an emerging field. Nano-Raman (TERS and SERS) approaches in near-field configuration allow the characterization of intrinsic properties of protein aggregates at concentration levels below nM, much lower than can be obtained with traditional analytical tools [4,5]. Trace detection and physicochemical characterization of protein aggregates are applied to study the chemical composition, secondary structure of proteins, and interaction with nanoparticles (NPs), such as TiO_2_ and ZnO [6,7].

Nanoparticles are widely used in biomedical sciences to develop new therapeutic pathways. However, relatively little is known about NP’s influence on amyloid proteins, such as α-synuclein (α-Syn) and β-amyloid aggregation and formation [8,9,10,11,12,13]. The hypothesized function of α-syn is related to vesicle trafficking and release. It is predominantly expressed in neuronal presynaptic terminals in the brain, being one of the most abundant proteins with an estimated intracellular concentration of 30–60 μM. Alpha-synuclein can self-polymerize into amyloid fibrils and the presence of such aggregates in the form of Lewy bodies (LBs) is a characteristic pathological feature of Parkinson’s disease and similar neurodegenerative disorders [14]. Aggregates produced from recombinant α-Syn in vitro are indistinguishable from those extracted from Lewy bodies of PD patients. While most cases of PD are of the late-onset sporadic type, there are rare cases of inherited early-onset PD caused by α-Syn gene triplications or by the presence of point mutations that are reported to increase the oligomerization and fibrillation propensities of α-Syn. Several in vitro studies have shown that the process of α-Syn aggregation in solution is concentration dependent and typically requires bulk protein concentrations above 100 μM. NPs’ interactions with proteins can damage the protein structure and cause toxicity. Alpha-synuclein misfolding and aggregation are often accompanied by β-amyloid deposition in some neurodegenerative diseases. Roberts et al. (2017) demonstrated that α-synuclein expression induced β-amyloid secretion and amyloidogenic processing in neuronal cell lines [15]. The β-amyloid, involved in Alzheimer’s disease, comes in several different molecular forms that collect between neurons. In the brain with Alzheimer’s disease, abnormal levels of this naturally occurring protein accumulate, forming aggregates and impairing cell function. The aggregation and accumulation of β-amyloid protein culminate with the formation of extracellular plaques. Some other works have demonstrated that beta-amyloid could trigger aggregation of alpha-synuclein in vitro [16] or that alpha-synuclein could inhibit beta-amyloid plaque formation [17]. It has also been found that aggregation was dependent on pH, peptide concentration, and time of incubation in an aqueous medium [12,18]. Therefore, examining interactions between different types of NPs and proteins is crucial to understanding the mechanisms of NP interactions and the development of safe and non-toxic nanoparticle-based commercial and therapeutic applications [19,20,21].

In this work, TiO_2_-NPs and ZnO-NPs were chosen because of their broad usage in different industry fields to produce papers, inks, skin care products, toothpaste, medicines, and food products. Therefore, the goal of this study is to gain a better understanding of the effect of ZnO-NPs, and TiO_2_-NPs on the conformation of α-Syn/βA protein, which is important for the application of NPs in vivo. NPs are small particles with dimensions between 1 and 100 nm. Due to their small size, NPs have the potential to penetrate the human body through various pathways, cross the blood-brain barrier, and promote fibrillation of amyloid proteins, and potentially cause neurotoxicity and neurodegenerative diseases [22].

In this work, we aimed to evaluate the interaction of characterized TiO_2_ and ZnO nanoparticles with α-synuclein and β-amyloid using Raman spectroscopy and to assess the influence of nanoparticles on amyloid proteins using multi-factor analysis.

## 2. Materials and Methods

The TiO_2_ and ZnO nanoparticles were obtained from Nanostructured and Amorphous Materials Inc., Los Alamos, NM, USA. 

Phosphate buffered saline (PBS), bovine serum albumin (BSA), water, hydrofluoric acid, silver nitrate, hydrogen tetrachloroaurate (III), ethanol, 2-propanol, β-amyloid fragment 1-40 (βA), and α-synuclein were obtained from Sigma Aldrich. All reagents and solvents used in this study were analytical grades.

### 2.1. Characterization of TiO_2_ and ZnO Nanoparticles

The compositions and structures of TiO_2_-NPs and ZnO-NPs were characterized by XRD, Raman spectroscopy, and UV-Vis spectroscopy, and nanoparticle size was measured by AFM. The TiO_2_-NPs characterization methodology and results are described in a previous author’s work [23]. 

The XRD measurements were performed on a SmartLab X-ray diffraction system with Bragg–Brentano geometry using Cu Kα radiation (0.15406 nm wavelength, voltage 30 kV). The step-scan covered the angular range 20–70 (2θ) in steps of 2θ = 0.01° (SmartLab X-ray Diffraction System, Rigaku Corporation, Tokyo, Japan). For the measurements, a compressed ZnO powder on the glass substrate (HirschmannLaborgerate microscope glass, 76 × 26 × 1 mm size)was used.

The Raman investigations were conducted on a WITec Raman spectrometer (with x100 high NA objectives, TE-cooled (down to −60 °C) electron multiplying CCD camera) at the excitation wavelength of 532 nm and a controlling laser power of 10 mW (WITecWissenschaftlicheInstrumente und Technologie GmbH, Ulm, Germany). The analyzed spectra were obtained by the average of 5 spectra with an acquisition time of 30 s. A drop (10 µL) of ZnO water solution (1 mg/1 mL) was placed onto the glass substrate (Carl Roth, 50 × 24 mm, #1), and then dried and measured.

The UV-Vis spectroscopy measurements were performed with a USB2000 fiber optic spectrometer (Ocean Optics, Orlando, FL, USA) operating at the wavelength range of 200–900 nm with an average of 5 spectra (acquisition time was 3 s). The measured ZnO water solution, using the quartz cuvettes (3 mL, CVD-UV1S type), had a weight concentration of 0.5 mg/mL.

Characterization of ZnO nanoparticle size was performed using atomic force microscopy (AFM) with Solver equipment from NT-MDT Inc. (Appendorn, The Netherlands) in tapping mode using commercial silicon cantilevers NSG11 with a force constant of 5 Nm^−1^. The ZnO-NPs water solution (of 1 mg/1 mL) was filtered through a 200 nm polyethersulfone pore membrane Chromafil PES-20/25, purchased from Macherey-Nagel (Dueren, Germany), was placed onto a glass substrate of 50 × 24 mm, #1 from Carl Roth, and then dried and assessed. Image analysis, data processing, and image acquisition were performed using the NOVA software from NT-MDT Inc. (Appendorn, The Netherlands).

### 2.2. Preparation of ZnO and TiO_2_ Nanoparticles

Weighted 5 mg of NPs was sonicated for 15 min in an ultrasonic bath along with 50 μL of 2-propanol. Then, 5 mL of ultrapure water or 5 mL of 18.8 M BSA was added into the suspension and sonicated for 15 min. From this mixture, 68 µg NPs/mL βA 1-40 or α-Syn (10 nM) suspension was prepared, which was sonicated for 15 min and additionally reacted for 3 h at 4 °C or 24 h at 37 °C. Prior to purification, ligand excess and/or organic solvent residues were removed by triple centrifugation/decantation (6000 rpm, 15 min.).

Then, capped NPs were redispersed by sonication in ultrapure water or PBS at 5 mL.

### 2.3. SERS Substrate Preparation

The preparation of silver SERS substrates was based on direct silver ions reduction using elemental silicon. Silicon wafers were cut into small pieces (1.5 cm × 1.5 cm), cleaned by immersing them in pure ethanol, and dried under the nitrogen flow. Hydrofluoric acid was diluted with water to the final concentration of 24%.

The silver precursor solution was prepared by diluting AgNO_3_ to the final concentration of 2.0 mM. Silicon wafers were immersed in prepared HF solution for 10 s, then immediately transferred to AgNO_3_ solution for 5 s, washed with deionized water, and dried under the nitrogen flow.

### 2.4. Sample Preparation for Raman and AFM Analysis

The pieces of glass coverslips were cleaned with piranha solution. Then, 25 µL of α-Syn/βA(10 nM) or 25 µL of NPs and α-synuclein suspension was dropped onto the surface and left to dry.

### 2.5. Samples Preparation for SERS Analysis

A silicon ring (0.9 cm diameter and 1 mm depth) was glued on a silver SERS substrate. Next, 60 µL of sample solution was dropped, covered with a glass coverslip, and left to dry for 1 h. The SERS analysis of a dry sample used the same sample as in suspension, except that the coverslip was removed and the sample was dried.

### 2.6. Sample Preparation for TERS Measurements

First, 25 µL of the prepared suspension of α-Syn coated nanoparticles (0.5 mg/mL) in deionized water was dropped onto a coverslip washed with piranha solution and dried. TERS spectra were measured with a confocal upright microprobe Raman/AFM system NT-MDT Spectra, (Appendorn, The Netherlands), equipped with a half-wave plate and polarizer for all experiments. ZnO-amyloid nanoparticles deposited on glass slides were excited using a 532 nm solid-state laser. Raman signals were collected from multiple spots on the substrate by 180° backscattering through the same microscope objective (long working distance 100× numerical aperture of 1.49) used to deliver the incident light. The AFM contact mode was used during the TERS measurements. The illumination laser spot position was adjusted to the end of the TERS tip using manual laser beam positioning and visual control with the installed CCD camera. Further control of tip pressure on the sample was accomplished by tuning the AFM feedback signal set point. The exposure time for the collection of all Raman spectra was 1 s. The tip used for TERS measurements was a bulk metal Ag probe made by electrochemical etching of Ag microwires using a Pt cathode, filled with a film of 8 M NH_4_NO_3_ aqueous solution and DC voltage 2.0 V, following the methodology developed by [24].

The tip testing was performed by measuring ten spectra per TERS probe, and averaged spectra were used for comparison.

### 2.7. Acquisition of Spectra and Analysis

Sixteen summary spectra from each isolate from different randomly selected spots on the SERS substrate were recorded. The measurements were taken without drying out the suspension drop, thus, limiting thermal damage. The single spectrum was acquired as a summary of spectra by scanning a 100 × 100-μm area during the SERS spectra acquisition procedure, i.e., one of the 16 summary spectra was acquired by adding up 100 × 100 spectra gathered in the test area. This was intended to optimize the Raman signal strength and to increase the reproducibility of summary spectra. The acquisition time for the Raman scattering signal was 20 s. The resulting spectra were processed using the Nova software from NT-MDT Inc. (Appendorn, The Netherlands) and the SpectraGryph 1.2.14 software (Dr. Friedrich Menges, Oberstdorf, Germany) [25] with the removal of background fluorescence, normalization to maximum amplitude, baseline correction of 5% coarseness. Vibrational band peaks were registered (position tolerance of 0.15%, peak finding threshold of 1.5%) and tentative peak assignments were determined based on comparable results in relevant literature sources.

Multivariate cluster and PCA analyses were performed to assess the TiO_2_-NPs and ZnO-NPs influence on amyloid conformation changes, using Raman processing software in the MATLAB (2012) environment (MathWorks, Inc., Natick, MA, USA) [26].

The reference spectrum for a single isolate used for PSA was produced from 16 experimentally acquired summary spectra as an average. 

## 3. Results and Discussion

### 3.1. Analysis of the Structure of TiO_2_ and ZnO Nanoparticles

#### 3.1.1. XRD Diffraction

The rutile phase was identified for TiO_2_-NPs, while an average crystallite size of 60 nm was obtained. The detailed characterization of TiO_2_ nanoparticles used in this work has been presented in a previous work by [23].

**Figure 1:**
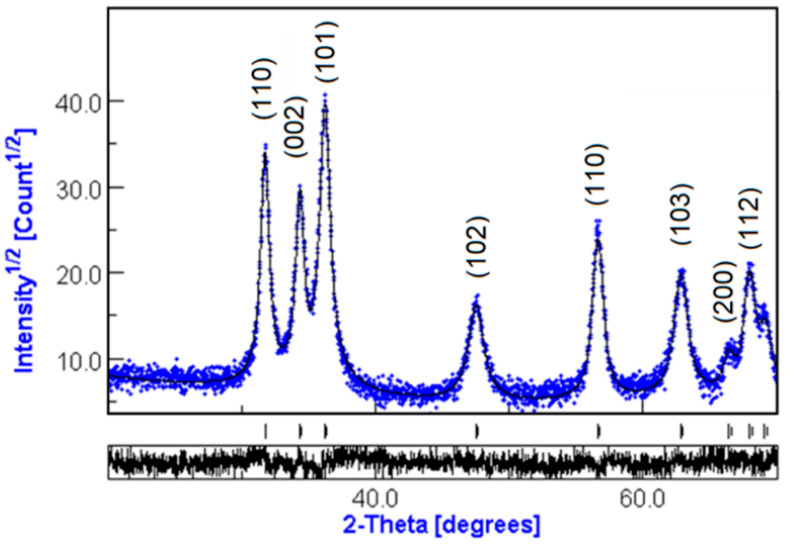
X-ray diffraction of ZnO nanopowders.

The phase composition of ZnO nanoparticles by XRD is presented in Figure 1. It was achieved by comparing the obtained results with the Crystallographic and Crystallochemical Database for Minerals and their Structural Analogues (http://database.iem.ac.ru/mincryst/index.php, accessed on 5 April 2022).

The analysis of XRD data was performed using the MAUD software (Material Analysis Using Diffraction, http://www.ing.unitn.it/~maud/ accessed on 10 May 2022). It is a general diffraction/reflectivity analysis program mainly based on the Rietveld method. The best fit was obtained in an isotropic model simulating planar defects with nanocrystal sizes of 21.9 ± 0.2 nm and a phase composition of 100% ZnO.

#### 3.1.2. Raman Spectroscopy Analysis

**Figure 2:**
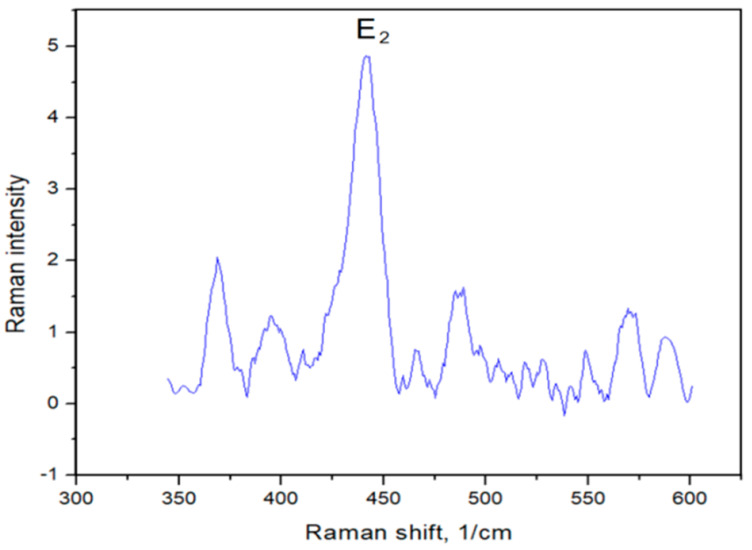
Raman spectra of ZnO nanoparticles.

The Raman spectrum of ZnO powder is presented in Figure 2.

For the removal of the background of the Raman spectra, the CrystalSleuth software (http://rruff.info/about/about_download.php, accessed on 20 April 2022) was used. The Raman spectrum of ZnO (blue line) is typical for ZnO nanostructures with the dominant E_2_ (high) phonon mode at 441 cm^−1^. The comparison with the database showed a strong dependence of ZnO phonon modes on the type of nanocrystals (particles, rods, tubes, etc.) and their size, and has been described in the work by [27].

#### 3.1.3. AFM Measurements of ZnO Nanoparticle Sizes

**Figure 3:**
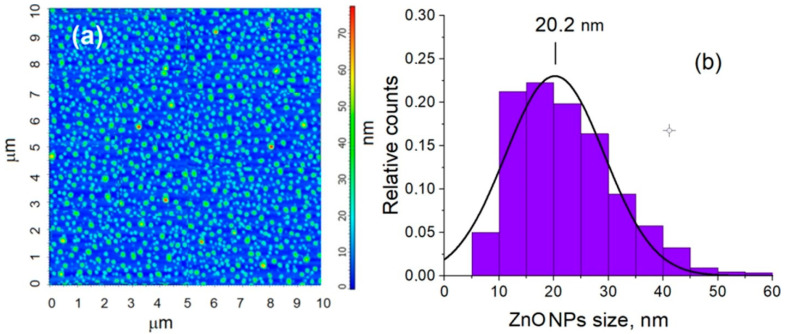
Atomic force microscopy-based analysis of ZnO nanoparticle solution: (**a**) Phase image of ZnO-NPs; (**b**) size distribution of ZnO-NPs.

The shapes of the ZnO nanoparticles were determined using the data analysis of AFM measurements (Figure 3). The NOVA software was used for the AFM analysis. To determine the size of the ZnO-NPs, the grain analysis function was used, and then the distribution of nanoparticle sizes was built, with the size of ZnO nanoparticles of 20.2 nm.

#### 3.1.4. UV-Vis Analysis

**Figure 4:**
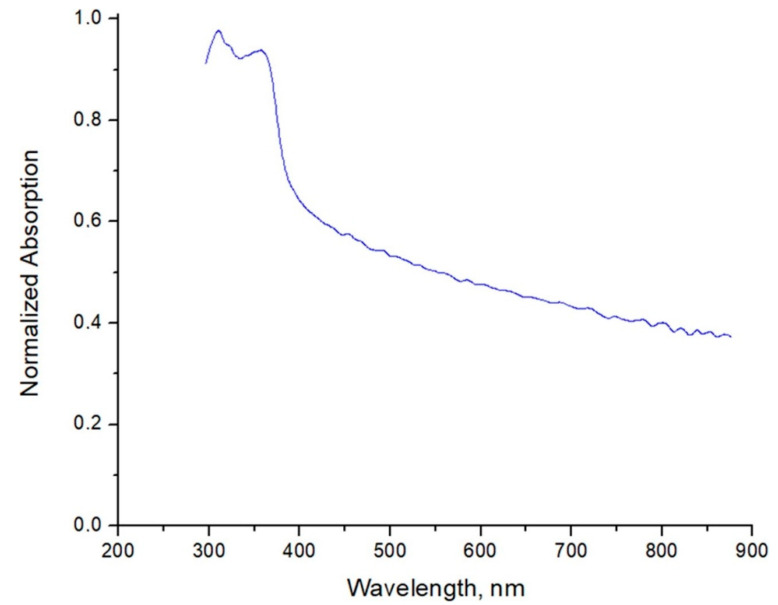
UV-Vis absorption spectra of ZnO-NPs water solution.

The UV-Vis absorption spectra of ZnO-NPs were investigated at room temperature in the range of 200–900 nm, as shown in Figure 4. The absorption peaks for ZnO-NPs were at λ = 320 nm, 377 nm, and very small at λ = 340 nm. A higher degree of redshift could be due to the bigger crystallite size. In general, the UV-Vis absorption spectra of the ZnO-NPs solution are typical ones and fit well with the results of the published works of other authors [28,29].

### 3.2. Investigation of the Effect of ZnO-NPs and TiO_2_-NPs on the Structure of βA 1-40 by SERS Spectroscopy at Temperatures of 4 °C and 37 °C

**Figure 5:**
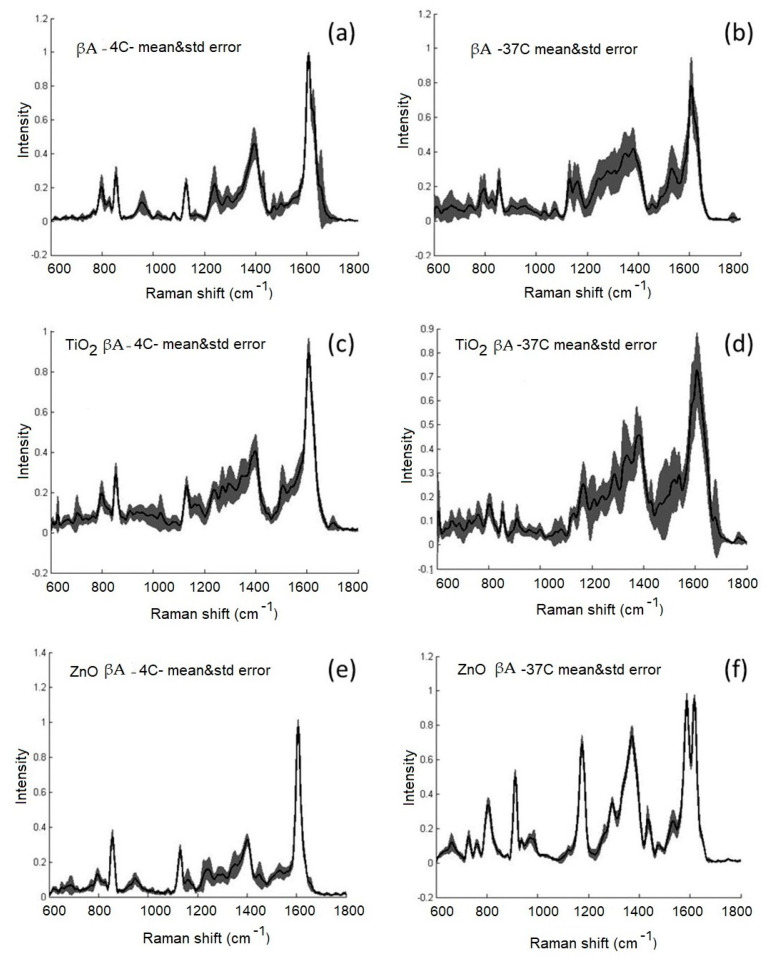
Averaged SERS spectra (*n* = 8–21) of suspensions of: (**a**,**b**) βA solution (10 nM); (**c**,**d**) TiO_2_-NPs coated with βA; (**e**,**f**) ZnO-NPs coated with βA, at 4 °C and 37 °C with standard errors.

ZnO-NPs and TiO_2_-NPs were coated with βA by incubating them for 3 h at 4 °C or 24 h at 37 °C in an aqueous solution. The βA had a monomeric structure in fresh solution at 4 °C temperature, and after incubation of the βA solution for 24 h at 37 °C, self-oligomerization was noticed. Similar results were also noticed by Howlettet et al. in microgram concentration of βA solutions, using 24–168 h at 37 °C incubation [12]. The effect of NPs on protein structure was assessed after incubation for 3 h at 4 °C and 24 h at 37 °C with 10 nM βA solutions. The protein not adsorbed on NPs was removed by centrifugation/decantation. The spectra of SERS showed signal patterns in the protein-rich structures (Figure 5). Significant peaks were detected for proline (890–920 cm^−1^), phenylalanine (1.000–1.030 cm^−1^), C–C stretching (proteins) (1.100−1.200 cm^−1^), amide III (1.200–1.300 cm^−1^), and amide I (1.600–1.700 cm^−1^). 

The average SERS spectra (*n* = 8–21) of suspensions of βA solution (10 nM), ZnO-NPs, and TiO_2_-NPs coated with βA at 4 °C and 37 °C with standard errors are shown in Figure 5. The average spectra show that when incubating α-Syn solution (*n* = 13) and preparing TiO_2_-βA NPs (*n* = 12) for 24 h at 37 °C, the variety of spectral changes increases, i.e., the spectra have a larger standard error. This may indicate that both, the protein in solution and the protein on TiO_2_-NPs at 37 °C, acquire various intermediate structures. The situation is different with ZnO-βA NPs (*n* = 14) after incubation at 37 °C. The deviations of the spectra from the average spectrum are small, which may indicate a more uniform structure of βA at all investigated points of the Ag SERS substrate. It is known that the amide I region is empowered for protein conformational change [30,31]. The Ag SERS substrate is the same in all experiments, ZnO-NPs or the ZnO-Ag combination likely has a stabilizing effect on a certain structure of the protein or the formation of a certain specific structure. Significant differences in the intensity, shape, and location of Raman and SERS spectra in the amide I region (1600–1700 cm^−1^) were found when comparing βA and ZnO-βA NPs or TiO_2_-βA NPs related to protein conformational changes and variations in their aggregation behavior. We identified the Raman peak in samples from βA of C=O stretch near 1650 cm^−1^ which is associated with the normal state of the protein. Similar conclusions were made after incubation with CuO nanoparticles, the relative cytotoxicity of α-synuclein oligomeric structures was more significant than α-synuclein amyloid alone [32]. In another study, NiO nanoparticle interactions with α-synuclein were tested and the obtained results indicated that the fabricated NiO NPs led to secondary structural changes of α-synuclein and accelerated protein amyloid formation through a significant increase in the kb and ka parameters as the homogeneous nucleation rate constant and the secondary rate constant, respectively [33]. SiO_2_ particles also induce oxidative stress and α-synuclein aggregation [34].

**Figure 6:**
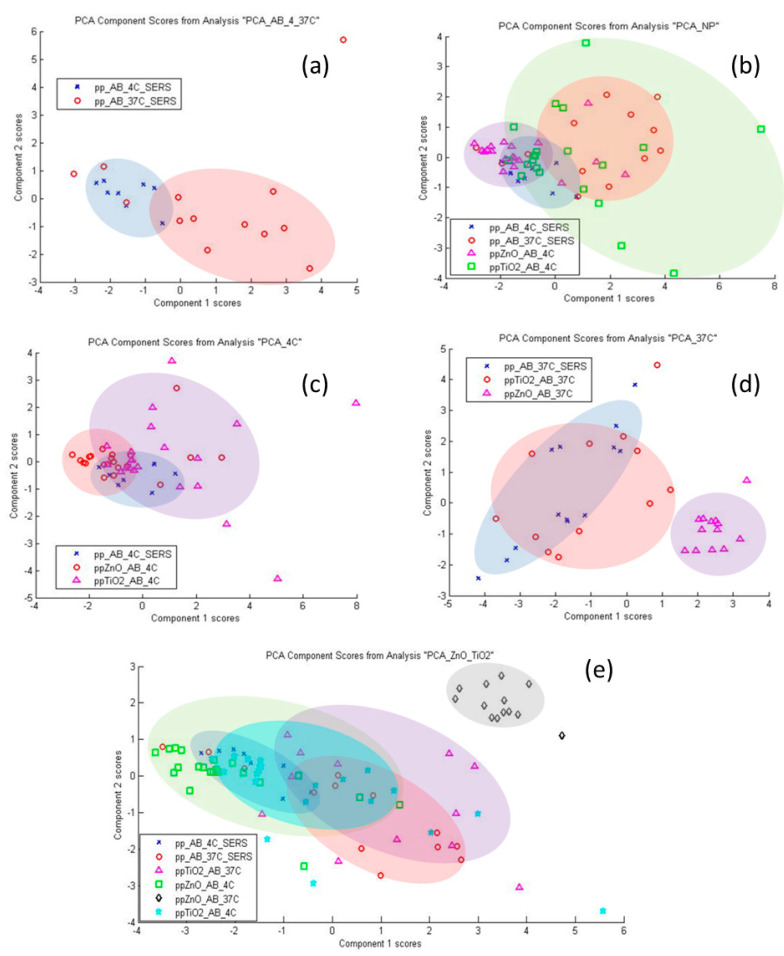
PCA analysis of SERS spectra (*n* = 8–21) of βA 1-40 solution (10 nM), TiO_2_-βA NP, ZnO-βA NP suspensions (0.5 mg NPs/mL): (**a**) Effect of 37 °C on βA structure; (**b**) effect of NPs and 37 °C on βA structure; (**c**) effect of NPs on βA structure (4 °C); (**d**) effect of NPs on βA structure (37 °C); (**e**) total temperature and NP effects on βA structure.

Biological materials such as α-synuclein can produce complex data with overlapping peaks in Raman spectroscopy or SERS. Principal component analysis (PCA) is an analysis method that optimizes the reduction of spectral data into a set of principal components (PCs) [33]. The first PC represents the groups with the most variations in chronological order, and the corresponding PCs describe the frequenc shifts in the spectral peaks.This method for Raman spectroscopy has been demonstrated as capable of detecting α-Syn aggregates in colon tissues and making it a promising tool for future use in PD pathology [34]. The obtained SERS spectra were investigated using PCA and the results are shown in Figure 6. Figure 6a (effect of incubation at 37 °C on the structure of βA, 11 PCs, 96.3% variance) shows that the spectra of incubated βA differ from the unincubated one in many points according to the first two components, but individual groups do not stand out. Figure 6b (comparison of the effect of NPs at 4 °C and 37 °C incubation on βA structure, 22 PCs, 95% variance) shows that NPs at 4 °C either do not cause changes in βA structure (dots overlap with βA) or those changes are similar to 37 °C incubations (dots overlap with βA incubated at 37 °C). Figure 6c (effect of NPs at 4 °C on βA structure, 19 PCs, 95.4% variance) shows that at 4 °C, part of the βA on NPs maintains the same structure as free βA, NP spots do not separate into separate groups. In Figure 6d (effect of NPs at 37 °C on βA structure, 18 PCs, 95.5% variance), a distinct group of spectra characteristic of βA-ZnO is distinguished. TiO_2_-NPs do not have a more significant effect on βA structure than incubation of the free protein at 37 °C. In Figure 6e (spectra of free βA and βA on NPs at 4 °C and 37 °C, 26 PCs, 95% variance), a group of spectra of βA-ZnO NPs prepared at 37 °C also stands out, which is also significantly different from βA-ZnO NPs prepared at 4 °C. There are no other separate groups.

The obtained results after coating and incubating ZnO-NPs and TiO_2_-NPs with βA by incubating them for 3 h at 4 °C or 24 h at 37 °C in an aqueous solution suggest that the protein binds to the surface of NPs to form a coating known as “protein corona”, which could affect the cytotoxic action of NPs and the level of NPs cytotoxicity in vivo. The impact of graphene oxide nanoparticles on the viability of Chinese hamster ovary and mouse hepatoma MH-22A cells with and without protein-albumin from bovine serum (BSA) has been previously described. The effect of GO nanosheets has been identified even at low concentrations of BSA (1–10%) [35,36,37]. The protein corona decoration strongly depends on the type of disease and proteins. Identical nanoparticles coated with varying protein corona decorations have exhibited significantly different cellular toxicity, apoptosis, and uptake [38]. Mirsadeghi et al. found that the protein corona formed a shell at the surface of gold NPs, regardless of their size and shape, reducing the access of βA to the gold inhibitory surface and affecting the rate of βA fibril formation, for example, anti-fibrillation potencies of various corona-coated gold NPs were strongly dependent on the protein source and their concentrations [39]. Other authors have also reported protein corona formation on the surfaces of NPs of amyloid proteins and that α-Syn could access the surface of NPs that were precoated with a protein corona, due to the dynamic nature of the protein corona proteins, and the protein corona reduced the accelerating effect of the NPs [10].

### 3.3. Investigation of the Effect of ZnO-NPs on the Structure of βA 1-40 by Raman and SERS Spectroscopy

**Figure 7:**
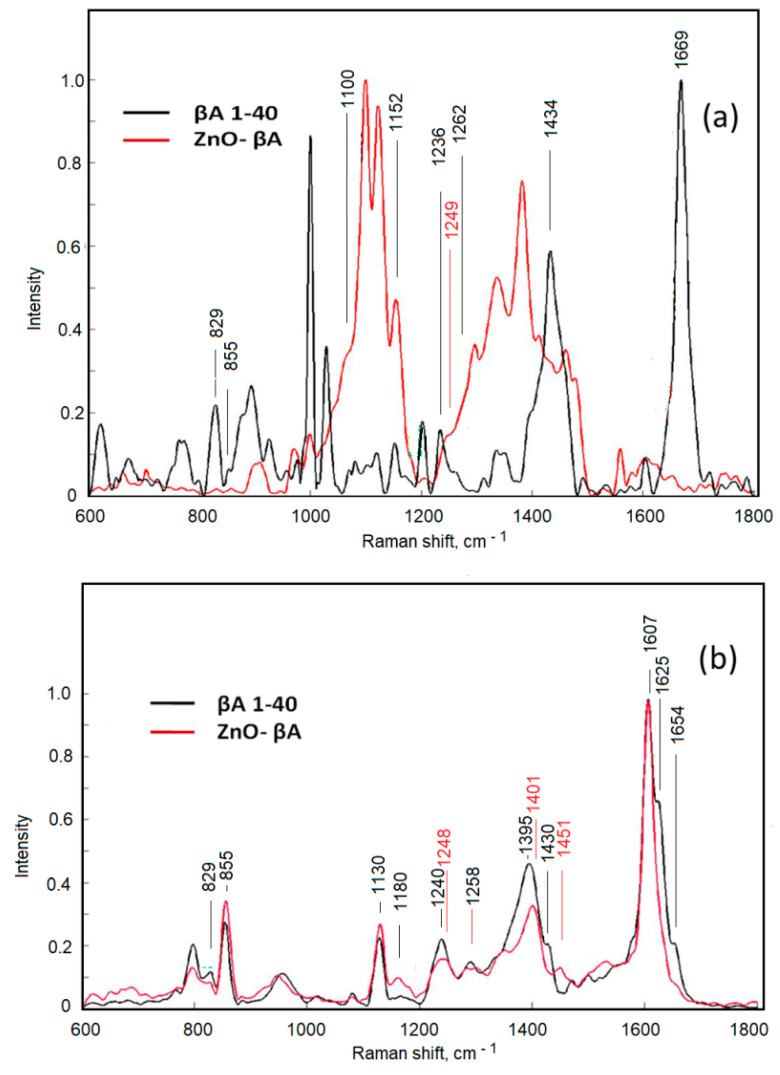
(**a**) Raman spectra; (**b**) SERS spectra (4 °C), of βA 1-40 (red) and ZnO- βA (black).

Raman spectroscopy has proven to be a unique, label-free, and non-destructive technique for the structural characterization of amyloidogenic proteins, their oligomers, and fibrils [40]. The structure of βA 1-40 was investigated by Raman spectroscopy and SERS (Figure 7) by incubating the protein for 3 h at 4 °C. The SERS and Raman spectra differ significantly, which may be influenced by the interaction of the protein with the Ag nanostructured surface and the reasons related to the different preparations of the samples for both analyses (identical preparation is not possible due to the lower sensitivity of the Raman spectroscopy method as compared with SERS). SERS spectra show smaller structural differences between βA 1-40 in solution and βA 1-40 on ZnO-NPs suspension (ZnO-βA) as compared with Raman spectra. The reason for this may be that the Raman spectra are recorded under different conditions than SERS, i.e., not in solution, but in drying drop conditions, when both βA 1-40 and ZnO-βA concentrations are at least 105 and 50 times higher than in SERS measurements, respectively. Therefore, the structure of the protein in the dry sample may differ from the structure in the 10 nM solution, due to the interaction with the Ag nanostructured surface and also due to the concentration effect. In the case of ZnO-βA nanoparticles, βA 1-40 concentrates on the NPs during droplet drying may promote fibrillation both due to the presence of NPs and the concentration effect (fibril-like formations on the NPs layer were observed by light microscopy in the Raman sample, AFM analysis is required for confirmation). The obtained results allow us to assume that it is not possible to record Raman spectra in conditions close to SERS analysis (in liquid) for studies of nanomolar concentrations of βA 1-40. The identifications of Raman and SERS spectra are presented in Table 1. The assigned spectra were listed along with their literature source.

In another study, β-amyloid fibril formation was observed and it was found that TiO_2_-NPs could promote β-amyloid fibrillation by shortening the nucleation process, which is the key rate-determining step of fibrillation [57]. Thus, the potential role of nanoparticles in treating the accumulation of amyloid-beta peptide in Alzheimer’s patients was reviewed.It was noticed that the nanoparticles from three different categories, i.e., polymer, lipid, and gold nanoparticles, could prevent the accumulation of β-amyloid during the efficient delivery of the drug to the cells to treat Alzheimer’s disease [58].

### 3.4. Investigation of the Effect of TiO_2_-NPs on the Structure of α-Syn by Raman and SERS Spectroscopy

To determine the effect of TiO_2_-NPs on the conformation of α-Syn, conformational changes were studied using different modifications of Raman scattering analysis (micro Raman, SERS, and TERS analysis methods).

TiO_2_-NPs were coated with α-Syn by incubating them for 4 h at 37 °C in a 10 nM α-Syn aqueous solution. α-Syn has a monomeric structure in a fresh solution at 4 °C temperature. The incubation of the α-Syn solution for 24 h at 37 °C was aimed at evaluating the self-oligomerization of α-Syn in a nanomolar concentration solution.

**Figure 8:**
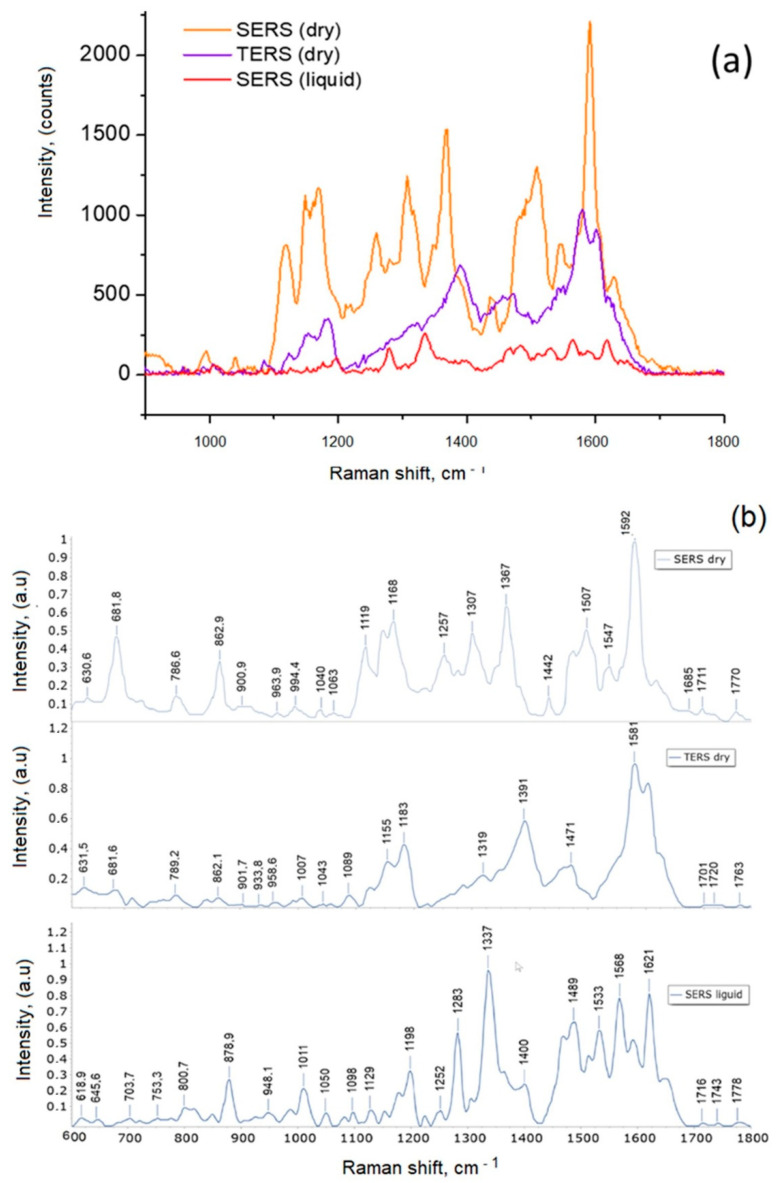
(**a**) Registered Raman spectra; (**b**) normalized spectra, of TiO_2_ -α-Syn NP.

Single recorded TiO_2_-α-Syn NP SERS (in suspension and dry sample) and TERS spectra, as well as normalized spectra after background correction are shown in Figure 8. For a more detailed analysis, correction and normalization of all registered spectra (12–20) and a comparative analysis of average spectra were performed. TERS spectra should theoretically be more uniform as compared with SERS spectra, which may depend on the heterogeneity of the nanostructured surface, but from the initial data, we can assume that TERS spectra are also quite heterogeneous. This may be related to the heterostructural coating of the TERS probe with silver crystals.

We characterized the structural features of α-synuclein in suspension and a dry sample. The Raman spectrum of the α-Syn solution (Figure 8) shows the vibration features of the amide III region near 1200–1300 cm^−1^ and the amide I region at 1600–1700 cm^−1^, which is attributed to the disordered protein conformations. Figure 8 reveals three representative types of SERS and TERS spectra of TiO_2_-α-Syn with the same settings for spectra registration using an acquisition time of 10 s and laser power of 0.25 mW. The amide I and the amide III bands of the SERS and TERS spectra exhibit prominent variations indicating the co-existence of different TiO_2_-α-Syn forms with various structures. Similar conclusions have been made by other authors after testing the SERS probe with α-synuclein in aqueous solutions [59].

The identification of the SERS and TERS spectrum analysis is presented in Figure 8b. Wave numbers at 1581–1621 cm^−1^ correspond to amide I, and those at 1252–1337 cm^−1^ correspond to amide III, indicating the α-synuclein species in α-helix and β-sheet structure. The amide I band at ~1685 cm^−1^ and the amide III band at 1220–1240 cm^−1^ are attributed to the hand coil structure consistent with its spontaneous Raman spectrum.

SERS using gold nanoparticles have been tested by other authors with an ultrathin α-synuclein shell into a tightly packed monolayer on glass support [60].

The presented results show that the TERS and SERS spectra of TiO_2_-α-Syn (dry) differ in the amide I/amide III ratios. The SERS spectra are more intense in the 600–1200 cm^−1^ region, also the SERS spectra recorded dry and in liquid are different, especially in the amide I region.SERS and TERS spectra confirm the changed conformation of α-Syn after incubation for 24 h at 37 °C. The SERS spectrum is richer than the TERS spectrum. Several studies have concluded that the process of protein aggregation can be triggered by various processes occurring on the surface of nanoparticles. Nanoparticles have electrical charges that stimulate nanoparticle adsorption, and their relatively large surface area can cause protein aggregation. It is well known that protein fibrillation is a nucleation-dependent mechanism and its initiation is stimulated by external factors. Nanoparticles act as a catalyst, stimulating the subsequent assembly of peptides into stable fibril aggregates. Nanoparticles can act as conventional catalysts by lowering the energy barrier between fibrils due to an increase in the population of anti-fibrillar aggregates. Nanoparticle surfaces can act as platforms for protein association, and this association induces significant changes in protein structure. The high temperature causes the proteins to unfold, leading to the formation of fibril-like structures. Higher protein concentrations lead to the formation of amyloid aggregates. In this work, non-toxic concentrations of nanoparticles [61] were used to investigate the effect of nanoparticles on α-synuclein aggregation. 

The obtained data showed that the interaction of α-synuclein with the surface of nanoparticles at such concentrations has a moderate affinity. However, protein affinity increases with increasing NP concentration, which is seen due to the interaction between protein corona and proteins in the solution and relatively higher exergonic reaction at higher protein concentrations. The obtained results at 4 °C and 37 °C show that α-synuclein aggregation intensifies with increasing temperature. The results obtained by this study showed the β-sheet structure formation of α-synuclein species, and as observed by authors [62], β-sheet structures led to aggregation.Summarizing the obtained research results, it can be stated that ZnO and TiO2 NPs influence the conformation of proteins and the consequences of that conformation, which are determined by the physico-chemical properties of both surfaces involved in the interactions, which is the basis and determines the type of complex formation. Therefore, by modifying the surface properties of nanoparticles, it is possible to influence the α-synuclein aggregation process and to prevent the aggregation of α-synuclein caused by nanoparticles and their interactions with biomacromolecules, which can be the cause of diseases.

## 4. Conclusions

The interactions between TiO_2_-NPs and ZnO-NPs on the aggregation kinetics of β amyloid fragment 1-40 and α-synuclein protein were investigated by surface-enhanced Raman spectroscopy (SERS) and tip-enhanced Raman spectroscopy (TERS). The characterization of crystalline phases of TiO_2_-NPs was evaluated by X-ray diffraction spectrum, atomic force microscopy, and UV-Vis spectroscopy. The interactions of ZnO-NPs with the amyloid proteins were investigated using SERS and TERS spectroscopy. The XRD analysis results showed that the phase composition of the nanoparticles was 100% ZnO with a crystallite size of 21.9 nm, while the AFM result showed that the peak particle size distribution was at 20.2 nm.

Our study showed that exposure of amyloid protein molecules to TiO_2_-NPs and ZnO-NPs after incubation at 37 °C caused the morphological structure of the molecules and stimulated molecule aggregation and even fibrillation. 

Significant differences in the intensity and location of active Raman frequencies in the amide I domain were found related to α-synuclein conformational changes and differences in their aggregation behavior. 

The chemometric assessment of the nanoparticle’s influence on amyloid conformation changes was performed using the principal component analysis (PCA) technique. The analysis results show that the spectra of incubated βA at 37 °C differ from the unincubated one in many points according to the first two components, but individual groups do not stand out. A comparison of the effect of NPs at 4 °C and 37 °C incubation on βA structure shows that NPs at 4 °C do not cause changes in βA structure.

This work enhances the current knowledge regarding the interactions of TiO_2_-NPs and ZnO-NPs with proteins and furthers a better understanding of the effect of ZnO-NPs and TiO_2_-NPs on the conformation of β-amyloid fragments 1-40 and α-Synuclein protein, which is important for the application of NPs in vivo.

## Figures and Tables

**Table 1:** Identifications of Raman and SERS spectra.

Wave Number (cm^−1^)	Assignment	References
829, 855	Tyrosine (Tyr)	[41,42]
1100	Alanine (Ala), νCC, νCN, νCO	[43,44]
1130	δ(CCH)	[45]
1152	νCC, Isoleucine (Ile)	[44]
1180	Tyrosine (Tyr), phenylalanine (Phe)	[41,46]
1236, 1240	Amide III, β-sheet	[47,48]
1248, 1249	Amide III	[46]
1258, 1262, 1654	α-Helix band, amide III	[46,49]
1395, 1401	Amide S	[50]
1430, 1434	Tryptophan (Trp)doublet, CH_2_, CH_3_ deformation	[46,51]
1451	CH_3_ and CH_2_ symmetric stretching, phenylalanine (Phe)	[52,53]
1607	Tyrosine (Tyr), phenylalanine (Phe)	[54]
1625	Tyrosine (Tyr), tryptophan (Trp)	[55]
1669	Amide I, β-sheet	[56]

## Data Availability

The data presented in this study are available on request from the corresponding author.
